# Spatial Variation in Educational Quality in Colombia Based on the Phenomena of Agglomeration and Academic Segregation

**DOI:** 10.3390/ejihpe12080072

**Published:** 2022-08-05

**Authors:** Geovanny Castro-Aristizabal, Gregorio Giménez-Esteban, David Arango-Londoño, Esteban Moreno-Cediel, Maribel Castillo-Caicedo

**Affiliations:** 1 The Research Group Sectoral Dynamics, The Universidad Autónoma de Bucaramanga, Avenue 42#48-11, Bucaramanga 680003, Colombia; 2 Department of Applied Economics, The University of Zaragoza, Pedro Cerbuna 12, 50009 Zaragoza, Spain; 3 Department of Natural Sciences and Mathematics, The Pontificia Universidad Javeriana Cali, Street 18#118-250, Cali 760031, Colombia; 4 Economy, Faculty of Social and Economic Sciences, The Universidad del Valle, Street 13#100-00, Cali 76001, Colombia; 5 Research Group Foresight and Strategic Thinking, The University of the Valley, Street 13#100-00, Cali 76001, Colombia

**Keywords:** educational quality, agglomeration, segregation, spatial autocorrelation, Moran’s Index, geo-visualization

## Abstract

This study seeks to measure the degree of agglomeration of educational quality in Colombia, based on the nonsocialization of the population that exhibits low educational quality, with the population that exhibits high educational quality, and thus determine how such agglomeration affects the phenomenon of academic segregation. To this end, we perform a spatial analysis of the educational quality in Colombia and of variables that may influence the phenomenon of educational agglomeration. The level of agglomeration in educational quality in Colombia is demonstrated by the calculation of the Moran’s Index, in which a result of 0.62 was obtained. High educational quality is concentrated in the Andean region, while low educational quality is agglomerated in the periphery of the country, in areas such as the Pacific region. A spatial regression model was carried out to measure the dependence of municipalities on their neighbors, and to determine the main socio-economic factors affecting the phenomenon of educational agglomeration in Colombia, finding that living conditions, unsatisfied basic needs and fiscal transparency all have an impact on the educational quality of the municipalities. It is also found that the number of homicides in the municipalities does not seem to have a significant relationship with education.

## 1. Introduction

The purpose of measuring the quality of an educational system is to determine the extent to which the goals and objectives proposed in the training and learning processes have been achieved. Therefore, the measurements through standardized tests or evaluations constitute a fundamental tool to obtain reliable and necessary information for the improvement of the educational systems. Based on these evaluations, it is possible to identify both the strengths and weaknesses of the system, which provides feedback for educational institutions and territorial entities [1].

Colombia has been applying the SABER tests and is participating in international evaluations of school performance to assess its educational system. These evaluations are applied at different educational levels and appraise different skills. Their results have been used in various studies that use multiple methodologies and that have analyzed the identification of factors associated with academic performance (see Castro et al. [2]; Ayala et al. [3]; Sánchez [4]), educational gaps estimates (see Castro et al. [5]; Cárcamo and Mola [6]) and efficiency studies (see OECD [7]). Results of the evaluations also serve as the basis for the calculation of the Synthetic Index of Educational Quality-ISCE in its Spanish acronym built by the Colombian Institute for the Promotion of Higher Education—ICFES—with which the classification of educational centers in Colombia is carried out.

Given the above, this work seeks to measure the degree of agglomeration of educational quality in Colombia, based on the calculation of the ISCE, for the year 2018, and to identify drawbacks that are the main socioeconomic factors that determine said agglomeration. In this way, we intend to identify how concentrated is the educational quality throughout the country in order to determine how the distribution of educational quality is causing academic segregation; that is, we intend to define how the agglomeration prevents students (the population) with a low level of school-related skills from socializing with students with a high level of school-related skills, and find the socioeconomic factors of the school environment and how they significantly influence said segregation.

The research problem is justified because the results obtained in previous studies focus on the effectiveness or the determinants of school performance, leaving aside a feature that has not been addressed much by studies oriented towards the measurement of educational quality in Colombia, the spatial location of the school. This feature has a relationship with the educational quality and could be originating a cluster in education. Likewise, research analyzing if the educational agglomeration causes the phenomenon of academic segregation is scarce.

The main results show, through the Moran’s Index, that there is a spatial correlation of 62%; that is, there is a high agglomeration in educational quality in Colombia, which is concentrated in the Andean region, encompassing the main cities of this region (Bogotá, Medellín and Cali), while low educational quality is concentrated in the periphery of the country, in areas such as the Pacific, Amazonian and eastern plains regions. In addition, the main determinants of this agglomeration were the urbanization rate [−], the unsatisfied basic needs [−] and the fiscal transparency of public institutions [+].

Now, from the literature review, research shows that the education is one of the most important pillars of society and is an essential factor in the development of countries. In Colombia, it is a fundamental right enshrined in the political constitution and is one of the most important issues in government plans, which constantly seek to increase the coverage and quality of education in the country. Education is a core tool to achieve a fair and equitable society.

From an economic point of view, the notion of agglomeration is linked to three concepts: (1) the economies of scale, (2) the size of the local market, and (3) the transportation costs [8]. It is important to mention that, in the case of services such as education, positive externalities have a fundamental effect on the quality of life of the population; thus, the economic approach must consider the spatiality to avoid, among others, the phenomenon of educational segregation which will be addressed in this project.

The influence of the context or neighborhoods on the quality of some services as education can have an effect on the future achievements of children. If the trajectory of the cities and their dynamics lead to agglomerations of educational institutions by areas with a clear segregation of socioeconomic status (a well-defined differentiation between rich and poor), and with a lack of access to better quality services because of their location, feelings of deprivation usually appear among heterogeneous individuals [9].

The literature focused on the Latin American context has identified key socio-economic conditions of the student’s environment that are linked with their academic success. Arcidiácono et al. [10] state that in Latin America, there is a clear public–private school segregation, and that in the last two decades, this segregation has deepened in the basic and middle educational levels. The authors notice the nonmigration of economically disadvantaged students to private schools that exhibit a higher educational quality, and they consider this worrying. There is a diminishing interaction between children from different socioeconomic strata. Residential integration is low as neighborhoods of medium and medium-high strata are separated, further pronouncing spatial segregation. Gaviria and Barrientos [11] identified that the characteristics of the parents have a significant influence on the performance of the students and that the positive relationship between the features of the educational facility and academic performance is limited to private schools which have kept a higher educational quality compared to public schools despite the increase in public spending. Giménez and Castro [12] focus on the differences between the students attending public and private schools, and they find that students of public schools have worse overall characteristics (mainly socioeconomic status), and this leads to notable differences in school results between public and private school students. Moreover, Giménez et al. [13] state that these differences can also be caused by peer effects, measured by schools’ average socioeconomic status or unsatisfied basic needs at the district level.

With respect to the characteristics of the environment where the schools are located, Giménez et al. [14] identify a strong nexus between the students’ performance and the social development of the districts where schools are located: the economic and educational dimensions of social development positively correlate with academic achievement. Rodríguez et al. [15] highlight that the quality of life and well-being are dimensions directly related to performance in many education systems. Additionally, Sullivan et al. [16] draw attention to the fact that urban schools tend to have better infrastructure and teachers than rural ones, contributing to an increase in its students’ achievement. Teacher qualification proves to be a key factor for learning [17,18]. Finally, Giménez and Barrado [19] point out that exposure to crime in the districts where schools are situated, measured by the homicide rate, has a negative and significant impact on academic achievement. This effect is particularly important in the case of students attending schools situated in districts with lower social development. Exposure to crime is also connected with the probability of suffering victimization by peer physical aggression at school, which can affect school results [20].

Based on this literature, the present study uses the level of urbanization, crime, economic development, quality of life and well-being as spatial factors linked to academic success. The existing literature identifies the factors that influence the quality of educational systems or illustrates the effect that agglomeration and segregation have on school performance or the education system. However, these studies *do not study the spatial effects of agglomeration and segregation by microeconomic units such as the country’s departments. For this reason, this work presents a novelty in its use of information and in its spatial treatment of it*.

## 2. Materials and Methods

The hypothesis is that the existence of agglomeration in the educational quality in Colombia and the presence of socioeconomic factors of the departments such as the number of homicides, the unemployment rate, thefts, among others, are generating a great deal of academic segregation, and this prevents the educational level in the country from increasing. To analyze this situation, we calculate the ISCE in the departments of Colombia for 11th-grade students for mathematics and language skills and for the year 2018, based on the methodology proposed by the ICFES for its estimation (see ICFES [21]). The study focuses on this section of the student population because the ISCE is applied to the basic and middle levels. Based on the results of the ISCE, the index is aggregated by departments.

Initially, the degree of spatial dependence of the ISCE by departments is evaluated using the Moran’s Index and testing various neighborhood structures (as queen and rook) and the spatial lags. Moran’s Index shows if departments with high ISCE are surrounded by other departments that also have a high ISCE (agglomeration).

Subsequently, the spatial econometric model for cross-sectional data is estimated, based on the methodology developed by Anselin et al. [22] and performing the aggregation of the score at the department level through the empirical Bayesian estimation of Lajaunie [23]. As inputs, we include variables on the individual characteristics of the student, his family and school. This information is collected from the ICFES databases and the form C600 of the National Administrative Department of Statistics—DANE by its Spanish initials. The contextual variables of the departments (homicide rates and socioeconomic stratum or socioeconomic level) are obtained from the same databases of the DANE and from the National Police. This allows us, on the one hand, to identify if the agglomeration of educational quality is generating academic segregation, and on the other hand, to establish which are the main socioeconomic determinants of this phenomenon.

To calculate the ISCE, we estimate the average SABER 11 test scores by department in Colombia for the subjects of mathematics and language in the year 2018, based on the individual scores obtained by students in the test. The SABER 11 test is designed and applied by the ICFES to seniors and assesses the average educational achievement of a student during his/her school life. Once the information has been added at the department level, it will be added to the mapping of the departments, using Geographic Information Systems—GIS, through software R and packages raster, rgdal and sp.

The ICFES, since 2015, has been calculating the ISCE which is composed by three aspects of secondary education: (1) progress, with a weighting of 40%, refers to the relative variation in the percentage of students with insufficient performance; (2) performance, with a weighting of 40%, considers the distribution of scores in SABER11 by quintiles; and (3) efficiency, which weighs 20% and corresponds to the student approval rate for the following school year. The ISCE ranges from 1, the lowest level of educational quality, to 10, the maximum level of educational quality. This study will be based on the methodology used by the ICFES for the calculation of the ISCE in Colombian departments for the year 2017 (see ICFES [21]).

### 2.1. Data

For the construction of the database, we used the statistical software R and the packages raster, rgdal, and sp. Based on the databases provided by the ICFES, where information on the results of the students on the SABER11 tests is collected for the two semesters of the year 2018, the database provided by the National Police, where there is data of the homicides that occurred in Colombia during the year 2018, and shape with the political map of Colombia, we began the georeferencing of the variables in the political map of Colombia. As a first step, we proceeded to join the two ICFES databases of the two semesters in which the tests were presented, for which we performed a match between both bases, so that the crossing between them was successful, and there was no cross of variables. Once the two bases were joined, we proceeded to take the necessary variables by means of a grouping between the code provided by the DANE for the municipality, and the variable to be studied; thus, groupings were created for the scores, the gender, the socioeconomic stratum, the educational level of parents, the household size, and other variables as having or not internet, TV or a computer. Likewise, from the database provided by the police, the same grouping was carried out for the number of homicides by each municipality.

Once the variables were prepared, they were added to the mapping of Colombia in order to carry out the spatial analysis. For this purpose, a cycle was built for each grouped variable, where the code provided by the DANE for the municipalities was related to the code of the municipalities provided by the cartography, thus successfully adding all the variables to said map in order to start with the descriptive and spatial analysis of them.

### 2.2. Descriptive Analysis

Table 1 shows some descriptive statistics of the areas evaluated by ICFES. The average of the scores exceeds 40 points; the highest scores are in mathematics and language, which receive special attention through this research. Regarding the global score, the average for Colombia stood at 238.3 points for the year 2018.

It is also observed that the proportion of women who took the exam for 2018 was higher (53.93%) than the proportion of men (46.06%); on the other hand, the most representative strata are stratum 2 and stratum 1, with a proportion of 37.23% and 33.24%, respectively. The most representative level of education among parents is complete high-school level. The most representative household size is 3 to 4 people, and in terms of the technological characteristics of the household, it is observed that most of the students have internet (59.72%), have a computer (58.78%) and have a television (77.25%). Regarding the characteristics of the territory, the average number of homicides by municipalities stands at 14.62 murders on average, while the average quality of life index by municipalities is 61.40, and the unsatisfied basic needs are 31.95 on average. 

**Figure 1:**
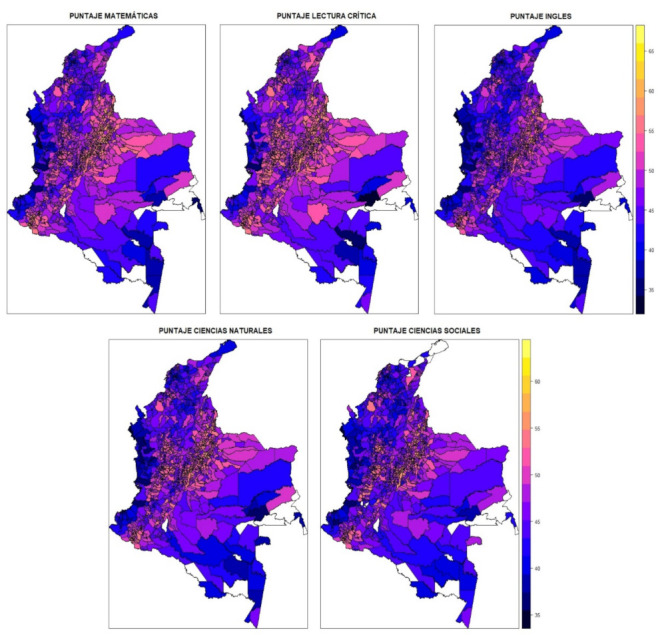
Spatial distribution of scores obtained by evaluated area. Note: PUNTAJE MAEMATICAS: Score Math; PUNTAJE LECTURA CRITICA: Score Reading; PUNTAJE INGLES: Score English; PUNTAJE CIENCIAS NATURALES: Score Natural Sciences; PUNTAJE CIENCIAS SOCIALES: Score Social Sciences. Color Scale: dark to light indicates lower to higher score.

Figure 1 shows the spatial distribution of the scores. When analyzing their geographical distribution, it can be seen that the highest scores, regardless of the evaluated subject, were obtained in the Andean Region of the country, while the lowest scores were obtained by students in the periphery of the country: Pacific region, Amazon region, part of the Eastern Plains and the Pacific Coast. The distribution presented may indicate the presence of agglomeration of educational quality; however, this hypothesis will be corroborated with formal tests. For contextualization, the reader can use the following link to the official physical–political map of Colombia in https://geoportal.igac.gov.co/sites/geoportal.igac.gov.co/files/geoportal/mapafisico.pdf (accessed on 1 June 2022).

### 2.3. Existence of Agglomeration

**Figure 2:**
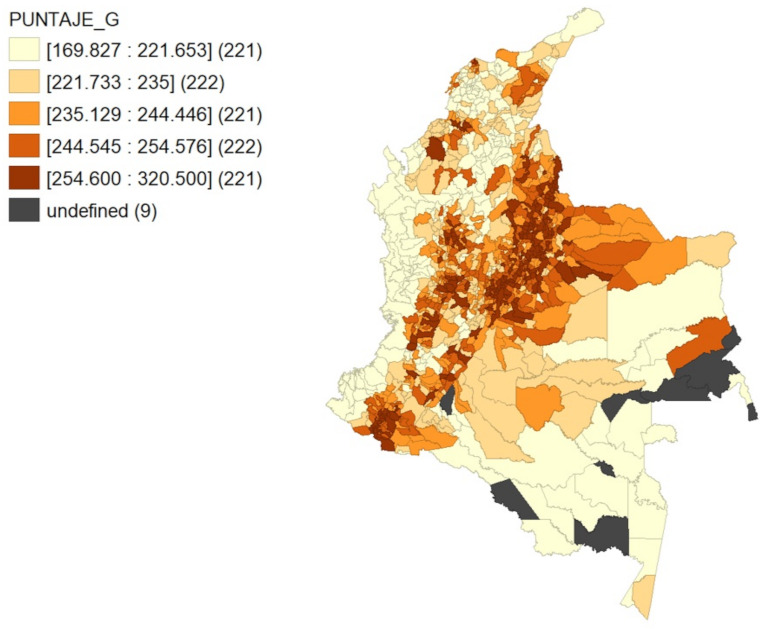
Spatial distribution of the Global Score. Note: PUNTAJE_G: Global Score. Color Scale: light to dark indicates lower to higher score.

The main hypothesis of our research is the existence of educational agglomeration in Colombia; to ensure this hypothesis, we resort to the Geoda program which is used for spatial data analysis, geo-visualization, spatial autocorrelation and modeling. With this program, the global test score is distributed in five quantiles, obtaining the map of Figure 2, in which the existence of educational agglomeration can be clearly observed. The high scores are concentrated in the Andean region of the country, while low scores are located in the peripheral area.

**Figure 3:**
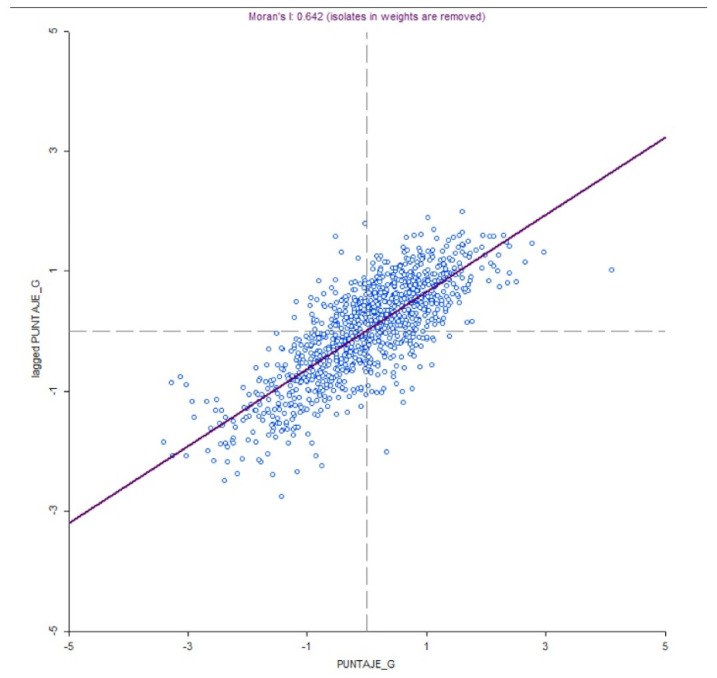
Calculation of the Moran’s Index for educational quality. Note: PUNTAJE_G: Global Score; lagged PUNTAJE_G: Lagging score.

However, to confirm the existence of agglomeration, we resort to the Moran’s Index which is used as a measure of spatial autocorrelation. It evaluates the degree to which an object is similar to other nearby objects; in this case, it measures the degree to which the educational quality of one municipality affects the educational quality of neighboring municipalities. The Moran’s Index can be classified as positive, negative or without spatial autocorrelation; a Moran’s Index very close to 1 indicates agglomeration, while one very close to −1 indicates segregation. When performing the analysis for this investigation, we obtained a result of 0.642 for Moran’s Index, which supports the hypothesis of the existence of agglomeration in Colombia (Figure 3).

### 2.4. Proposed Model

For the construction of the model, variables related to the socio-economic conditions of the student’s environment, aggregated by municipality, are considered. We evaluated the analysis of factors such as stratum, education of parents, household characteristics such as household size, technology indicators (internet, television or computer possession), and the number of homicides in each municipality. However, these characteristics are already included in variables calculated by the DANE that explain the socio-economic characteristics of the municipality. The explaining factors that we use have been highlighted as academic success determinants in Section 2.

**Urbanization rate**: It is defined as the proportion of the population of a municipality that lives in the urban area compared to the total of the inhabitants of the municipality. This rate reflects the pressure that the municipal seat exerts on the territory of the municipality, on its natural resources, and on the characteristics that are typical of the municipality. A high figure for this rate indicates a higher level of human development.

**Quality of Life Index (QLI)**: Provided by DANE, from the National Quality of Life Survey, which is based on a methodology that links the factors evaluated in this survey with satisfaction with life of each individual. This survey characterizes the living conditions of the municipalities with variables that are related to housing, education, health, labor force, child care, income and expenditure, while in terms of households, it considers variables such as possession of goods and the perception the head of household has of the living conditions of his or her household. The higher the QLI of the municipalities, the higher their educational quality is expected to be.

**Unsatisfied Basic Needs (UBN)**: This variable provided by DANE aims to determine to what extent the basic needs of the population are covered. It helps to establish the population qualified as poor. The dimensions considered in this variable are inadequate housing, critically overcrowded housing, housing with inadequate public services, housing with economic dependence, and housing with children who do not attend school. The higher the UBN measure of the municipalities, the lower the educational quality is expected to be.

**Homicides by municipality**: This variable measures the number of intentional homicides in each municipality, and it is provided by the National Police. It is expected that the more homicides that occur in a municipality, as an index of violence, the lower the educational quality in that municipality.

**Fiscal Performance Index (FPI)**: Variable provided by the National Planning Department (DNP). Its objective is to evaluate the performance of the territorial administrations in the aspect of public finances. It shows the fiscal behavior of the country’s municipalities and departments, to identify the strengths and weaknesses of each administration’s financial capacity. It is expected that the higher the fiscal transparency of each municipality, the higher its educational quality.

According to the possible components that could model the educational quality in Colombia and the socio-economic variables that may affect it, the statistical model is proposed:

(1) y=ρWy+Xβ+ε 

where y is a vector (N × 1) of the N observations of the dependent variable, W represents the spatial weight matrix, ρ is the autoregressive parameter that captures the intensity of the interdependencies between the N sample observations (spatial autocorrelation coefficient), Wy is the spatial delay of the variable y, X is a matrix of k exogenous variables, and ε is a term for white noise disturbance. This methodology allows us not only to locate possible clusters of educational quality but also to detect if there is any process of dissemination of educational quality. Thus, the statistical model is as follows:

(2) $$Score_{ij}=\rho W_{ij}Score_{ij}+\beta_{o}+\beta_{1}URBR_{ij}+\beta_{2}QLI_{ij}+\beta_{3}UBN_{ij}+\beta_{4}Homicides_{ij}+\beta_{5}FPI_{ij}+\varepsilon_{ij}$$

where *Score* is the scores obtained in each of the areas evaluated by the ICFES (Mathematics, Critical Reading, Natural Science, Social Science, English and the Global Score), *β*0 is the functional intercept of the model, $\rho W_{ij}$ is the spatial lag operator, which is formed by defining the corresponding neighbor of each department as a new column with a nonzero element, ($W_{ij}$) as a matrix of spatial weights, having as characteristics a positive and nonstochastic matrix, with each variable spatially delayed, URBR is the urbanization rate of each municipality, *QLI* is the quality life index, UBN is the unsatisfied basic needs, Homicides is the number of homicides per municipality, FPI is the fiscal performance index and $\varepsilon_{ij}$ is the error term associated with the model.

## 3. Results

Six regression models with a spatial lag of the dependent variable are considered, one for each area evaluated of the ICFES and for the global score, with the following results.

Table 2 shows the estimated coefficients of each of the regressions with their respective level of significance (*p*-value). All models explain more than 40% of the educational quality (see Appendix A). 

The model whose dependent variable is the global score as an approximation to the educational quality in the country in 2018 exhibits a significant spatial lag for the regression; the obtained coefficient for the urbanization rate implies that as the urbanization rate increases by one percentage point, the educational level is expected to decrease by approximately 9 points, while QLI and FPI showed the expected (positive) relationship; i.e., if the QLI in the municipalities increases by one point, the educational quality is expected to increase by approximately 0.8 points, and for the fiscal development index, if the administrative transparency of the municipalities is increased by one point, the educational quality is expected to increase by about 0.16 points. On the other hand, as expected, as unmet basic needs increase, the overall score decreases by approximately 0.25 points. Finally, the homicides variable was not significant in this model.

Mathematical and critical reading skills are fundamental to people’s education. After carrying out the respective econometric regressions, it is found that the spatial lag is significant in both. The quality-of-life index, the unsatisfied basic needs and the fiscal performance index also turn out to be significant and with the expected sign, while the number of homicides is not significant for any of the regressions. It is interesting to note that when the regression is performed with the mathematics score as a dependent variable, the results indicate a negative relationship; that is, if the number of homicides in the municipalities increases by one person, it is expected that the educational quality (taking the mathematics score as an approximation) decreases by approximately 0.00017 points; however, as stated, this variable is not significant for any of the tested models.

## 4. Discussion

This paper identifies spatial agglomeration as a fundamental factor of segregation. Agglomeration conditions school academic performance (relationship estimated between the urbanization rate and SABER11 test scores) and, therefore, affects the success of the Colombian educational system. These results coincide with those found by Arango et al. [24].

The results of the paper are novel because other studies dealing with the phenomenon of segregation and its relationship with development focused on factors other than spatial agglomeration. Specifically, other factors identified by previous studies were as follows:

1. Gender: this is attributed to the process of socialization to which young people are subjected from the moment they are born. When they grow up, they make their own decisions based on what is expected of them because of their gender identity [25,26]. Stereotypes are generated that impact their formal educational process [27,28,29]
2. Ethnicity: resulting from the concentration of a population minority. In the case of Colombia, the indigenous population presents low school performance [30,31] and suffers labor discrimination [32].
3. The level of school competencies is also defined as school segregation. It is caused by the distribution of students due to their individual or personal characteristics. This has an impact on social inequality and limits the development possibilities of the student body, particularly the most vulnerable students [33,34], leading them to poor school performance [35].
4. Social discrimination in which the student body is “separated” by their origins or social classes. At this point, findings show that private schools concentrate on upper and upper-middle-class students and have even more qualified teachers, which reflects higher academic performance in students, widening educational inequalities between public and private schools, and negatively affects the most vulnerable groups [12,36,37,38,39].

The concentration of high educational quality in the Andean region of Colombia is a result that has been pointed out by prior research. Bonilla [40] highlights that the Andean region is where both the higher salaries and the higher per capita income of the country concentrate. At the same time, the Pacific Region is where income inequality has grown the most [40]. With respect to the population distribution in Colombia, in the second half of the 20th century and the beginning of the 21st century, the country has gone from being an eminently rural country to an urban one, with most of the people migrating from the rural to the urban world settling in the Andean region [39]. In addition, the average household size has decreased, especially in the Andean region, as the number of children in urban households is lower than in rural ones [40]. These demographic transformations have been accompanied by important changes in the education sector, and in the distribution of education spending [41]. The most qualified teachers are concentrated in the Andean region of the country, which has led to better academic results for the Andean students [41].

## 5. Conclusions

The results obtained show that there is indeed educational agglomeration in Colombia, a situation that is corroborated by the high Moran’s Index obtained (0.64). The spatial analysis shows that high educational quality is concentrated in the Andean Region, while low educational quality is concentrated on the periphery of the country.

The methodology proposed here also made it possible to locate clusters of educational quality, as well as to detect a process of dissemination of education. The results show significant evidence of spatial dependence at the municipal level, so we can conclude that geographic location is an important factor in educational quality in the country. 

The presence of limiting (neighboring) municipalities is fundamental in explaining the educational quality in the country because it is found that most of the municipalities with low educational quality are surrounded by municipalities that also show low educational quality, and on the other hand, most of the municipalities with high educational quality are surrounded by municipalities that also have high educational quality.

Exogenous socio-economic factors greatly affect the educational quality in Colombia. Improving living conditions, addressing unmet basic needs and having greater fiscal transparency would help to increase the educational quality and decrease the phenomenon of agglomeration. The number of homicides in the municipalities is not a significant variable for the model; however, it is a proxy variable for the level of violence in the country, and greater peace of mind for a society in this regard could positively affect the educational quality.

The urbanization rate proved to have a negative effect on the educational quality in the country, a result that was not expected. However, this result can be explained because the urbanization rate is oriented to issues related to urban infrastructure and its functioning, necessary for the structural organization of cities, rather than to the infrastructure reflected in the educational institutions which may explain this negative relationship. 

Another possible way of modeling the relationships would be to create explanatory variables by means of principal component analysis. This may allow a better fit of the model in less computation time, and the results could be more conclusive. However, this analysis does not consider the endogenous factors that may cause low educational quality, such as the quality of teachers, infrastructure, and school resources, among others; thus, we suggest the realization of a case analysis for the municipalities with the highest levels of poor educational quality.

## Figures and Tables

**Table 1:** Descriptive Statistics.

Variables	Percentage/Average	[95% CI]	Spearman Coefficient
***Test scores (By area)***			
Global Score	238.31	[237.13–239.50]	
Mathematics Score	47.96	[47.68–48.25]	
Critical Reading Score	49.91	[49.70–50.13]	
Natural Science Score	47.46	[47.22–47.70]	
Social Science Score	45.51	[45.27–45.75]	
English score	47.03	[46.80–47.26]	
***Gender***			
Male	46.06%%	[45.93–46.19]	−0.0169
Female	53.93%	[53.80–54.06]	−0.0062
***Strata***			
Stratum 1	33.25%	[33.12–33.38]	−0.1008
Stratum 2	37.23%	[37.10–37.36]	0.1878
Stratum 3	21.12%	[21.01–21.23]	−0.0102
Stratum 4	5.21%	[5.151–5.273]	−0.1433
Stratum 5	2.00%	[1.967–2.044]	−0.1613
Stratum 6	1.17%	[1.143–1.202]	−0.1734
***Education of the mother***			
None	2.29%	[2.256–2.337]	−0.3046
Incomplete primary schooling	15.41%	[15.32–15.51]	−0.0414
Complete primary schooling	10.98%	[10.90–11.06]	0.0868
Incomplete high school	15.01%	[14.92–15.11]	−0.0244
Complete high school	25.61%	[25.49–25.73]	0.0277
Incomplete technical or technological	2.98%	[2.936–3.027]	0.0532
Complete technical or technological	9.99%	[9.911–10.07]	0.0290
Incomplete undergraduate studies	2.40%	[2.364–2.447]	0.0941
Complete undergraduate studies	10.89%	[10.81–10.97]	0.0281
Graduate studies	2.42%	[2.382–2.465]	0.2097
Not applicable	0.16%	[0.158–0.180]	−0.0160
Does not know	1.80%	[1.765–1.837]	0.0189
***Education of the father***			
None	3.59%	[3.539–3.639]	−0.2759
Incomplete primary schooling	19.59%	[19.47–19.69]	−0.0007
Complete primary schooling	10.96%	[10.87–11.04]	0.1195
Incomplete high school	13.95%	[13.85–14.03]	−0.0239
Complete high school	22.06%	[21.94–22.16]	−0.0021
Incomplete technical or technological	2.01%	[1.978–2.053]	0.0537
Complete technical or technological	6.76%	[6.696–6.831]	0.0264
Incomplete undergraduate studies	1.93%	[1.901–1.976]	0.0671
Complete undergraduate studies	9.48%	[9.406–9.564]	0.0014
**Variables**	**Percentage/Average**	**[95% CI]**	**Spearman correlation coefficient**
Graduate studies	2.21%	[2.178–2.258]	0.1558
Not applicable	1.03%	[1.006–1.061]	0.1071
Does not know	6.38%	[6.314–6.446]	0.0843
***Characteristics of the household***			
***Household size***			
1 to 2	7.46%	[7.399–7.539]	0.0166
3 to 4	48.39%	[48.26–48.52]	0.1026
5 to 6	32.06%	[31.93–32.18]	−0.0235
7 to 8	8.59%	[8.524–8.673]	−0.1885
9 or more	3.58%	[3.425–3.522]	−0.2552
Has access to the internet	59.72%	[59.58–59.85]	0.1141
Does not have access to the internet	40.27%	[40.14–40.41]	−0.0848
Has a TV	77.25%	[77.13–77.36]	0.0033
Does not have a TV	22.74%	[22.63–22.86]	−0.0289
Has a computer	58.78%	[58.65–58.91]	0.1294
Does not have a computer	41.21%	[41.08–41.34]	−0.1143
***Characteristics of the municipality***			
Homicides	14.62	[10.48–18.77]	−0.0247
Urbanization Rate	0.41	[0.396–0.425]	0.1091
Quality of life index	61.40	[60.75–62.05]	0.4404
Fiscal Performance Index	56.91	[56.22–57.60]	0.3371
Unsatisfied Basic Needs	31.95	[30.74–33.17]	−0.5554

**Table 2:** Significance of regression coefficients.

	Global	Mathematics	Critical Reading	Social Science	Natural Science	English
Spatial lag	0.0944 ***	0.1367 ***	0.0596 ***	0.0784 ***	0.1362 ***	0.0905 ***
URBR	−9.330 ***	−2.0311 *	−1.5246 ***	−2.1885 **	−2.1392 **	0.0800
QLI	0.8073 ***	0.1594 ***	0.1715 ***	0.1730 ***	0.1394 ***	0.1452 ***
UBN	−0.254 ***	−0.0585 ***	−0.0431 ***	−0.0519 ***	−0.0501 ***	−0.0479 ***
Homicides	0.00465	−0.00017	0.00127	0.00152	0.00065	0.00269
FPI	0.1684 ***	0.0354 ***	0.0276 ***	0.0331 **	0.0323 **	0.0493 ***

Signif. codes: 0 ‘***’ 0.001 ‘**’ 0.01 ‘*’ 0.05 ‘.’ 0.1.

## Data Availability

The data presented in this study are available on request from the corresponding authors.

## Appendix A

**Table A1:** Specification of the regression models.

	Global	Mathematics	Critical Reading	Social Science	Natural Science	English
R^2^	0.4839	0.4138	0.5471	0.5060	0.4143	0.5256
Breusch–Pagan test (*p*-value)	0.00030	0.00000	0.00000	0.00247	0.00038	0.35307
Significance of spatial dependence	0.00000	0.00000	0.00045	0.00012	0.00000	0.00000
